# Validation of a novel molecular assay to the diagnostic of COVID-19 based on real time PCR with high resolution melting

**DOI:** 10.1371/journal.pone.0260087

**Published:** 2021-11-22

**Authors:** Beatriz Iandra da Silva Ferreira, Natália Lins da Silva-Gomes, Wagner Luis da Costa Nunes Pimentel Coelho, Vanessa Duarte da Costa, Vanessa Cristine de Souza Carneiro, Rafael Lopes Kader, Marisa Pimentel Amaro, Lívia Melo Villar, Fábio Miyajima, Soniza Vieira Alves-Leon, Vanessa Salete de Paula, Luciane Almeida Amado Leon, Otacilio Cruz Moreira

**Affiliations:** 1 Real Time PCR Platform RPT09A, Laboratory of Molecular Biology and Endemic Diseases, Oswaldo Cruz Institute/ Fiocruz, Rio de Janeiro, Brazil; 2 Laboratory of Technological Development in Virology, Oswaldo Cruz Institute/ Fiocruz, Rio de Janeiro, Brazil; 3 Laboratory of Viral Hepatitis, Oswaldo Cruz Institute/ Fiocruz, Rio de Janeiro, Brazil; 4 Laboratory of Molecular Virology, Oswaldo Cruz Institute/ Fiocruz, Rio de Janeiro, Brazil; 5 University Hospital Clementino Fraga Filho, Federal University of Rio de Janeiro, Rio de Janeiro, Brazil; 6 Oswaldo Cruz Foundation (Fiocruz), Branch Ceará, Eusebio, Brazil; Cairo University, EGYPT

## Abstract

The emergence of the COVID-19 pandemic resulted in an unprecedented need for RT-qPCR-based molecular diagnostic testing, placing a strain on the supply chain and the availability of commercially available PCR testing kits and reagents. The effect of limited molecular diagnostics-related supplies has been felt across the globe, disproportionally impacting molecular diagnostic testing in developing countries where acquisition of supplies is limited due to availability. The increasing global demand for commercial molecular diagnostic testing kits and reagents has made standard PCR assays cost prohibitive, resulting in the development of alternative approaches to detect SARS-CoV-2 in clinical specimens, circumventing the need for commercial diagnostic testing kits while mitigating the high-demand for molecular diagnostics testing. The timely availability of the complete SARS-CoV-2 genome in the beginning of the COVID-19 pandemic facilitated the rapid development and deployment of specific primers and standardized laboratory protocols for the molecular diagnosis of COVID-19. An alternative method offering a highly specific manner of detecting and genotyping pathogens within clinical specimens is based on the melting temperature differences of PCR products. This method is based on the melting temperature differences between purine and pyrimidine bases. Here, RT-qPCR assays coupled with a High Resolution Melting analysis (HRM-RTqPCR) were developed to target different regions of the SARS-CoV-2 genome (RdRp, E and N) and an internal control (human RNAse P gene). The assays were validated using synthetic sequences from the viral genome and clinical specimens (nasopharyngeal swabs, serum and saliva) of sixty-five patients with severe or moderate COVID-19 from different states within Brazil; a larger validation group than that used in the development to the commercially available TaqMan RT-qPCR assay which is considered the gold standard for COVID-19 testing. The sensitivity of the HRM-RTqPCR assays targeting the viral N, RdRp and E genes were 94.12, 98.04 and 92.16%, with 100% specificity to the 3 SARS-CoV-2 genome targets, and a diagnostic accuracy of 95.38, 98.46 and 93.85%, respectively. Thus, HRM-RTqPCR emerges as an attractive alternative and low-cost methodology for the molecular diagnosis of COVID-19 in restricted-budget laboratories.

## Introduction

Severe acute respiratory syndrome coronavirus 2 (SARS-CoV-2) is a positive-sense single-stranded RNA virus [1] causative of coronavirus disease 2019 (COVID-19), the airborne-transmitted respiratory illness responsible for the COVID-19 pandemic [2]. Since its emergence in December 2019, until July 5^th^, 2021, the WHO (World Health Organization) has reported over 183 million cases and about 4 million deaths from COVID-19 [3]. Compared to the last WHO weekly report, the highest numbers of new cases were reported in India, Brazil, United States of America, Turkey and Argentina. The majority (~ 80%) of COVID-19 patients have mild symptoms or are asymptomatic, and about 20% of cases may require hospitalization due to breathing difficulty. The mortality rate of COVID-19 has been estimated at around 3% [4].

The availability of the complete SARS-CoV-2 genome during the initial phase of the pandemic facilitated the development of specific primers and standardized laboratory protocols for COVID-19 molecular diagnostic [5, 6]. The protocols for the first real-time RT-qPCR assays, targeting the SARS-CoV-2 RNA-dependent RNA polymerase (RdRp), envelope (E) and nucleocapsid (N), were first published in January 2020 [7]. After that, the WHO produced a technical manual [8], where seven in-house RT-PCR assays developed by CDC-China, Pasteur Institute-France, CDC-USA, NIID-Japan, Charité- Germany, HKU-Hong Kong and NIH-Thailand were recommended. All the recommended protocols are TaqMan-based methods that use probes designed for the different regions of the viral genome. Of these assays, only one uses an internal human control (RNAse P gene, CDC-USA protocol) that serves as a control for the nucleic acid extraction, which aids in successfully validating a truly negative result. Using these various approaches, a variety of molecular diagnostic kits were produced and validated in record time. Despite the fast development and deployment of these molecular diagnostic kits, production could not keep up with the global demand for diagnostic kits. This decrease in molecular diagnostic kit availability resulted in increased kit prices, making them cost prohibitive for developing countries. Therefore, the development of low-cost alternative diagnostic technologies would be beneficial for current and future medical needs.

The post-PCR High Resolution Melting (HRM) analysis is a high-sensitive and cost-effective molecular biology technique based on the Melting Temperature (Tm) difference between purine and pyrimidine bases that can be used for the detection of mutations, polymorphisms and epigenetic differences (quantification of methylation status of CpG sites) in double-stranded DNA samples [9]. qPCR followed by HRM analysis (HRM-qPCR) based methodologies have been reported for the detection and genotyping of different pathogens, such as virus, bacteria and protozoa in clinical samples [10–13]. This approach has the capability to combine different sets of primers specific to multiple nucleic acid targets within the genome of a single pathogen or multiple pathogens and/or an internal control within the same assay by using HRM analysis in differentiating the unique Tm of each target. The HRM-RTqPCR master mix uses fluorophore probes, instead of fluorescent probes, making this approach more cost-effective than other commercial molecular diagnostic kits. Furthermore, this approach provides molecular PCR-based diagnostic results coupled with the power of sequencing at a low cost.

In this study, real-time RT-PCR assays coupled with High Resolution Melting analysis were developed for different regions of the SARS-CoV-2 genome (RdRp, E and N) and an internal control (human RNAse P gene). The assays were standardized and validated using synthetic sequences from the viral genome and clinical specimens (nasopharyngeal swabs, serum and saliva) of patients with clinical suspicion of COVID-19 from different states within Brazil, in comparison to TaqMan RT-qPCR assays, as gold standard. Therefore, we developed a highly sensitive and specific assay for COVID-19 diagnosis as an alternative to commercially available TaqMan assays, which are not widely available to developing countries due to their high costs, hindering access to accurate and time-sensitive diagnostic results.

## Methods

### Ethics statement

This study was approved by the ethical committee of Clementino Fraga Filho University Hospital, from Federal University of Rio de Janeiro (UFRJ), CAAE number 31240120.0.0000.5257. All patients or a member of their families signed the consent form.

### Clinical samples

In this study, a panel of 42 nasopharyngeal swab, 12 serum and 11 saliva samples from 65 patients with severe or moderate COVID-19, admitted during the acute phase of infection (up to 14 days after symptom onset) were included. Forty-five patients from the state of Rio de Janeiro and twenty patients from the state of Ceará were included in this study. Fifty-one samples were positive and fourteen were negative for SARS-CoV-2 using the RT-qPCR TaqMan assay targeting the N region of the viral genome. The inclusion criteria for consideration of the sample was the presence of clinical symptoms consistent with COVID-19 during hospital admission (cough, headache, fever, anosmia and/or ageusia) and at least one positive SARS-CoV-2 laboratorial analysis. The patients presenting clinical symptoms consistent with COVID-19 but with a negative RT-qPCR TaqMan assay result were included as negative patients. Patients under 18 years old, pregnant women, and cancer patients were excluded from this study.

Nasopharyngeal samples were collected by inserting a swab through the nostril of the patient and rotating the swab along the nasal wall until reaching the nasopharynx. Once contact with the nasopharynx was made, the swab was gently rotated for 2–3 seconds. The swab was placed into a 5 mL tube containing 3 mL of viral transport medium (VTM. LABORCLIN, Pinhais (Paraná), Brazil). In the laboratory, the swab-containing tubes were vortexed vigorously, and the VTM was transferred to 1.5 mL tubes and stored at -80°C pending RNA extraction.

Saliva samples were self-collected from patients in a sterile dry tube which was closed after the saliva was placed inside. Clinical staff disinfected the outside of the tube using a 10% bleach solution after collecting the tubes from the patient to prevent contamination. Samples were delivered to the laboratory using a sample transport box.

Serum samples were collected from patient blood, 5 mL of patient blood, harvested in a BD Vacutainer Plus Plastic Serum tube that was undisturbed for 30 min at room temperature allowing the blood to clot. The blood clot was separated from the serum through centrifugation, 1,000 x g for 10 min at 4°C, and serum was collected and stored at -80°C until use.

### Viral RNA extraction and cDNA synthesis

RNA was extracted from 140 μL of the nasopharyngeal swab VTM (Virus Transport Media, LABORCLIN, Pinhais (Paraná), Brazil. Cat. No.: 511259. ANVISA Registry number: 10097010180), serum or saliva, using the QIAamp Viral RNA Mini Kit (Qiagen, Hilden, Germany), according to the manufacturer’s instructions. Briefly, 560 μL of prepared Buffer AVL, containing carrier RNA, was pipetted into a 1.5 mL microcentrifuge tube. VTM, 140 μL, was added to the microcentrifuge tube and mixed by pulse-vortexing for 15 s. The tube was then incubated at room temperature for 10 min and briefly centrifuged. Post-centrifugation, 560 μL of 100% ethanol was added to the sample and mixed by pulse-vortexing for 15 s. The tube was briefly centrifuged and 630 μL of the solution within this tube was carefully transferred to the provided column (coupled in a 2 mL collection tube). The column was centrifuged at 6000 x g for 1 min and placed into a clean 2 mL collection tube. Buffer AW1, 500 μL, was added into the column and centrifuged at 6000 x g for 1 min. The column was placed into a new 2 mL collection tube after centrifugation. Buffer AW2, 500 μL, was added to the column and centrifuged at full speed (20,000 x g) for 3 min. The column was placed into a new collection tube after centrifugation and centrifuged at full speed for 1 min. To collect RNA, the column was placed in a clean 1.5 mL microcentrifuge tube and 60 μL of Buffer AVE, at room temperature, was added to the column and incubated at room temperature for 1 min. The tube was then centrifuged at 6000 x g for 1 min. RNA concentration and quality was estimated using a Nanodrop ND-2000 (ThermoFisher, Waltham (Massachusetts), USA), by the absorbance assessment at 260 and 280 nm, and stored at -80°C until use.

For the two-step HRM-RTqPCR assays, reverse transcription was performed using 5 μL RNA, from the collected patient samples, using the SuperScript III First-Strand Synthesis System (Invitrogen, Waltham (Massachusetts), USA) and 1 μL of the equimolar mixture of 2019-nCoV_N2-R, nCoV 1 _IP4-14146Rv, E_Sarbeco_R2 and RP-R primers at 2 μM each.

### Real time RT-PCR with TaqMan assays

All patient samples were analyzed for SARS-Cov-2 N2 and human RNAse P targets using a commercial One-Step RTqPCR TaqMan kit, 2019-nCov CDC RUO Kit (IDT DNA, Newark (Nova Jersey), USA. Cat. No. 10006625), according to the manufacturer’s instructions. RT-qPCR was carried out in a 10 μL reaction containing 2 μL RNA, 5 μL Promega Go-Taq Probe-1-Step-RT-qPCR System [2X] (Promega, Madison (Wisconsin), USA), 0.2 μL GoScript RT mix for 1-step RT-qPCR, 0.75 μL Mix Primer-Probe FAM/BHQ (N2 or RNAse P) TaqMan, 0.25 μL CXR Reference Dye and 1.8 μL ultrapure water. Real-time PCRs were carried out on the Applied Biosystems Quantstudio 3 Real-Time PCR System (ThermoFisher, Waltham (Massachusetts), USA) using the following cycling conditions: 15 min at 50 ºC, 10 min at 95°C, followed by 40 cycles of 15 s at 95 ºC and 60 s at 55 ºC. Fluorescence was collected after each cycle at the annealing/extension step. All samples were run in duplicate, and the threshold was set to 0.02 for analysis.

### Primer selection

To select the primers for the HRM-RTqPCR assays (Table 1), sequences were further analyzed using the OligoAnalyzer Tool (IDT DNA, Newark (Nova Jersey), USA), available at https://www.idtdna.com/calc/analyzer. The specificity of the selected primers was previously validated by the CDC (USA), Institut Pasteur (Paris), and Charité-Universitätsmedizin Berlin (WHO) [8]. Additionally, we conducted an *in-silico* specificity analysis for these primers by using the primer-BLAST search tool (https://www.ncbi.nlm.nih.gov/tools/primer-blast). The criteria for selection of primer pair sequences allowed up to 4 mismatched nucleotides (none in the 3´ end) in the complete set of the NCBI database (non-redundant sequences) and resulted in SARS-CoV-2 and RNAse P gene related-sequences. None of the primer sets used in this study showed genomic cross-reactivity with the human genome, other viruses, or any probable interfering genome in the BLAST database analysis.

**Table 1:** Primers, linearity and melting curve parameters for SARS-CoV-2 and human RNAse P amplification.

Target	Primers	Origin	Sequences	Amplicon size	Dinamic extension (Linearity)	PCR efficiency	Melting temperature of the PCR product
**N**	2019-nCoV_N2-F	CDC-USA	5’-TTA CAA ACA TTG GCC GCA AA-3’	67 bp	10^6^–10 copies/μL	109.7%	80.8°C
	2019-nCoV_N2-R		5’-GCG CGA CAT TCC GAA GAA-3’				
**RdRp**	nCoV_IP4-14059Fw	Pasteur	5’-GGTAACTGGTATGATTTCG-3’	107 bp	10^6^–10 copies/μL	105.4%	83.5°C
	nCoV 1 _IP4-14146Rv		5’-CTGGTCAAGGTTAATATAGG-3’				
**E**	E_Sarbeco_F1	Charité	5’-ACAGGTACGTTAATAGTTAATAGCGT-3’	113 bp	10^6^–10 copies/μL	88.3%	77.6°C
	E_Sarbeco_R2						
			5’-ATATTGCAGCAGTACGCACACA-3’				
**RNAse P**	RP-F	CDC-USA	5’-AGA TTT GGA CCT GCG AGC G-3’	65 bp	10^6^–10 copies/μL	95%	86°C
	RP-R		5’-GAG CGG CTG TCT CCA CAA GT-3’				

To select the appropriate primers for the development of the HRM-RTqPCR assays, primers designed for COVID-19 molecular diagnostic testing described by the WHO [8] were evaluated based on the Tm similarity between each primer set (paired forward and reverse primers), low probability of hairpin structures, self-dimer and hetero-dimer formation tendency, small amplicon size (below 120 bp), and PCR efficiency. The selected primers were 2019-nCoV_N2-F and 2019-nCoV_N2-R (CDC, USA), nCoV_IP4-14059Fw and nCoV 1 _IP4-14146Rv (Institut Pasteur, France), E_Sarbeco_F1 and E_Sarbeco_R2 (Charité, Germany) and RP-F and RP-R (CDC, USA) targeting the N, RdRp and E regions of the SARS-Cov-2 genome and Human RNAse P gene, respectively (Table 1).

### Real time RT-PCR with high resolution melting

For the HRM-RTqPCR assays, cDNA was analyzed using SARS-CoV-2 N2, RdRp and E target sequences. The human RNAse P gene was used as an internal control. All reactions were performed by using a final volume of 10 μL; 2 μL [5X] HOT FIREPol EvaGreen HRM Mix (No ROX) (Solis Biodyne, Tartu, Estonia), a master mix containing the EvaGreen double-stranded DNA-binding dye, 200 nM of the 2019-nCoV_N2 F and 2019-nCoV_N2 R primers, or 300 nM of the nCoV_IP4-14059Fw and nCoV_IP4-14059 Rv primers, or 200 nM of the E_Sarbeco_F1and 300 nM E_Sarbeco_R2 primers (Table 1), or 300 nM of the RP-F and 300 nM RP-R primers, 2 μL cDNA and ultrapure water to reach a final volume of 10 μL. Real-time PCRs were carried out on the Applied Biosystems Quantstudio 3 Real-Time PCR System (ThermoFisher, Waltham (Massachusetts), USA) using the following cycling conditions: 10 min at 95°C, followed by 40 cycles of 15 s at 95ºC and 60 s at 58 ºC (N and RdRp targets) or 60°C (E and RNAse P targets), where fluorescence was recorded after each cycle. Melt curve stage conditions were: 10 s at 95°C, 60 s at 60°C, 15 s at 95°C (with fluorescence being recorded at a 0.025°C/s rate) and 15 s at 60°C. Threshold was set at 10,000 for analysis.

### Controls and analysis

The following positive and negative controls were used: low positive control (synthetic template of single-stranded DNA containing the SARS-CoV-2 N, RdRp and E genes (GenBank NC_045512.2) at 200 copies/μL), very low positive control (synthetic template of single-stranded DNA containing the SARS-CoV-2 N, RdRp and E (GenBank NC_045512.2) at 20 copies/μL) (S1 Table), and a negative template control, where ultrapure water was loaded at the plate instead cDNA. In addition, ultrapure water was also used instead the clinical specimen as a negative control for RNA extractions, in each extraction batch. QuantStudio Design and Analysis Software v1.5.1 (Applied Biosystems, Waltham (Massachusetts), USA) was used to analyze amplification plots and Ct values. High Resolution Melt Software v.3.2 (Applied Biosystems, Waltham (Massachusetts), USA) was used to differentiate between the high-resolution melting curves and obtain Melting Temperature (Tm) values. This software utilizes an improved clustering algorithm that accurately distinguishes between control and variant genotypes, allowing the user to input the expected number of clusters for increased sensitivity for difficult SNP genotyping.

### Analytical validation

The analytical validation (Dynamic Extension, Precision, Reproducibility) assays were performed using synthetic single-stranded DNA molecules containing select regions of the N, RdRp and E genes belonging to the SARS-CoV-2 isolate Wuhan-Hu-1 (GenBank NC_045512.2). For the linearity assay, a 1:10 serial dilution of the synthetic DNA was performed ranging from 10^6^ to 1 copies/μL in TE buffer. For the precision assay, three concentrations of the synthetic SARS-CoV-2 templates were used (12, 10 and 8 copies/μL). Forty technical replicates were assayed in each concentration by same operator in the same day, and results were compared. For the reproducibility assay, the same three concentrations of the synthetic sequences, at 12, 10 and 8 copies/μL, were also used. Amplification was performed for the 40 technical replicates of each template concentration, divided in two consecutive days, by the same operator.

### Statistical analysis

All experiments were performed at least in two technical replicates. Data distribution was evaluated by the Shapiro-Wilk normality test. Student’s t-test or Mann–Whitney Rank Sum test was used to analyze the statistical significance of the observed differences between two groups (according to the parametric or nonparametric distribution of the values, respectively) and Kruskal-Wallys one-way ANOVA followed by Dunn’s post hoc test was used to analyze the differences between three groups, with SigmaPlot for Windows version 12.0 (Systat Software, Inc, San Jose, California). In addition, the Bland-Altman method comparison was performed to compare the Ct values for SARS-CoV-2 detection using the RT-qPCR TaqMan or HRM assays, using the same software. Values of sensitivity, specificity, PPV, NPV, Diagnostic accuracy and Cohen’s kappa coefficient were calculated using the Open-Source Epidemiologic Statistics for Public Health (OpenEpi software V3.01), available at www.openepi.com. Results were expressed as means and standard deviations, and differences were considered significant if *P*< 0.05.

## Results

### Analytical validation of HRM-RTqPCR assays

**Fig 1:**
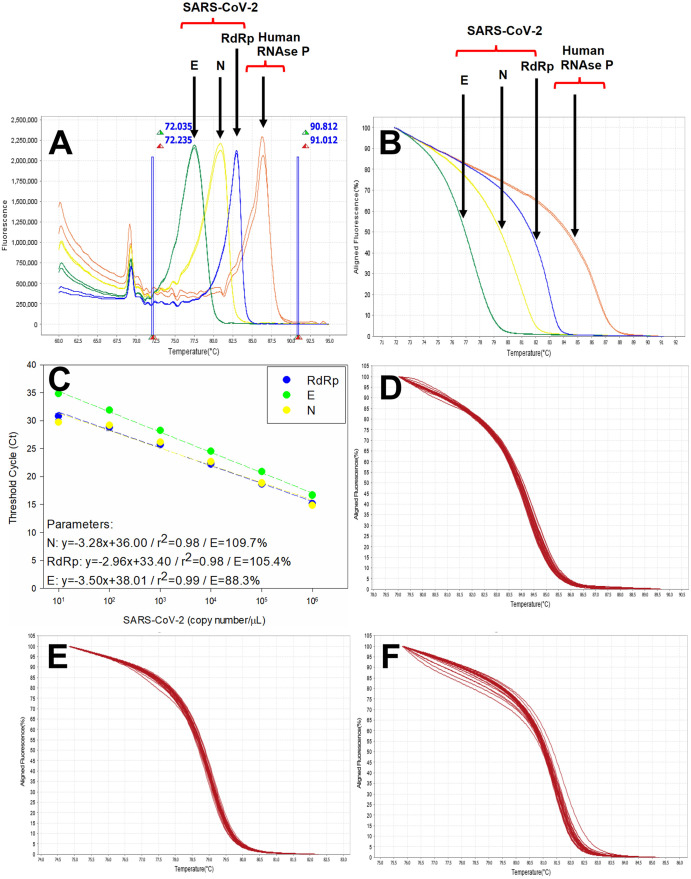
HRM-RTqPCR assays for SARS-CoV-2 E, N and RdRp targets. **A**. Derivative HRM curves for SARS-CoV-2 E (green), N (yellow) and RdRp (blue) and Human RNAse P (red) targets. The vertical blue lines divide the Pre-melt, Melt and Post-melt regions. **B**. Normalized HRM curves for SARS-CoV-2 E (green), N (yellow) and RdRp (blue) and Human RNAse P (red) targets. **C**. Dynamic extension (linearity) for the SARS-CoV-2 E (green), N (yellow) and RdRp (blue) gene target amplification. **D**. Normalized HRM curves for SARS-CoV-2 N region from COVID-19 clinical samples. **E**. Normalized HRM curves for SARS-CoV-2 RdRp region from COVID-19 clinical samples. **F**. Normalized HRM curves for SARS-CoV-2 E region from COVID-19 clinical samples.

After standardization, the HRM-qPCR assays were initially validated using synthetic DNA sequences of SARS-CoV-2 (GenBank NC_045512.2). A single and sharp peak at the derivative HRM curve was generated for each target, with Tm 80.8, 83.5, 77.6 and 86°C for N, RdRp, E and RNAse P, respectively (Table 1 and Fig 1A). In accordance with the observed Tm values, the pre-melt and post-melt regions of the HRM analysis were set before 72 and after 90.8°C, respectively (Fig 1A). Thus, the normalized HRM graph shows four distinguished curves that could be collectively analyzed (Fig 1B). The analysis for the standard curves produced by the serial dilution of the synthetic controls showed a dynamic range between 10^6^ and 10 copies/μL for the N, RdRp and E SARS-CoV-2 gene targets (Table 1 and Fig 1C). With the PCR efficiencies between 88.3 and 109.7% and the low Ct difference between the three targets, HRM-RTqPCR could be used to quantify viral load with great accuracy. In addition, it was possible to observe the same approximate limit of detection (LOD)–the lowest concentration detected in the linearity assay, around 10 copies/ μL, between the three gene targets of SARS-CoV-2.

The same normalized HRM curve profile was observed in comparison to the synthetic controls, with few differences between the clinical samples (RNA extracted from nasopharyngeal swab, serum or saliva) (Fig 1D–1F to the N, RdRp and E targets, respectively), validating the use of the HRM curves to confirm the product of amplification of SARS-CoV-2 in the positive samples.

Following analytical validation, the precision of HRM-RTqPCR assays was evaluated. To determine the precision of the HRM-RTqPCR assay, three concentrations around the estimated LOD (10 copies/μL) were selected: 12, 10 and 8 copies/μL of the synthetic SARS-CoV-2 templates. For the N, RdRp and E gene targets, forty technical replicates were assayed in each concentration, and the results were compared (Table 2). For the N gene target, 100% positive amplification was observed in the three concentrations, with Ct means (± Standard Deviation) of 29.80±0.51, 30.44±0.43 and 30.57±0.53, and coefficients of variation of 1.73%, 1.40% and 1.74%, respectively. For the RdRp gene target, 97.5% of positive amplification was observed in the three concentrations, with Ct means of 30.85±0.51, 30.77±0.99 and 30.70±0.83, and coefficients of variation of 3.74%, 3.20% and 2.71%, respectively. Finally, for the E gene target, 97.5%, 87.5% and 97.5% positive amplification was observed in the 12, 10 and 8 copies/μL, with Ct means of 28.87±0.46, 28.41±0.36 and 28.70±1.05, respectively. Within the E gene target assay, the coefficients of variation were 1.60%, 1.27% and 3.65%, respectively. Even with these observed slight differences, the coefficient of variation was lower than 5.0% between all gene targets and concentrations tested, highlighting the high precision of these assays.

**Table 2:** Precision of HRM-RTqPCR assays targeting SARS-CoV-2 E, N and RdRp. SARS-CoV-2 templates were assayed in 40 technical replicates, at 12, 10 and 8 copies/μL.

Parameter	Sample concentration		
	12 copies/μL	10 copies/μL	8 copies/μL
**N target**			
Positive samples	100%	100%	100%
Ct mean (± SD)	29.80 (0.51)	30.44 (0.42)	30.57 (0.53)
Coefficient of variation	1.726%	1.404%	1.735%
**RdRp target**			
Positive samples	97.5%	97.5%	97.5%
Ct mean (± SD)	30.85 (1.16)	30.77 (0.99)	30.70 (0.83)
Coefficient of variation	3.743%	3.202%	2.706%
**E target**			
Positive samples	97.5%	87.5%	97.5%
Ct mean (± SD)	28.87 (0.46)	28.41 (0.36)	28.70 (1.05)
Coefficient of variation	1.595%	1.267%	3.646%

SD: Standard deviation.

**Fig 2:**
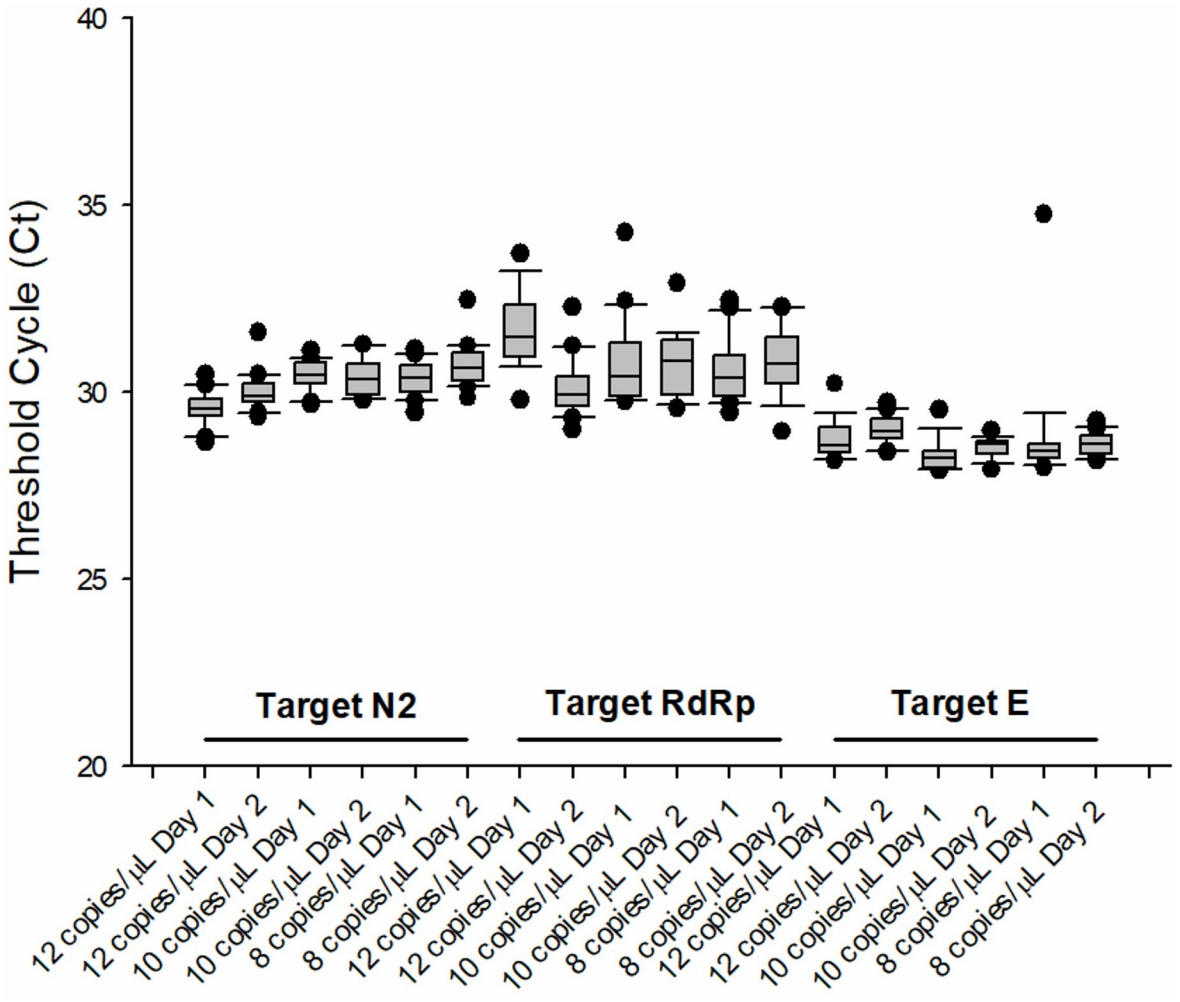
Reproducibility of HRM-RTqPCR assays targeting SARS-CoV-2 E, N and RdRp. SARS-CoV-2 templates were assayed in 40 technical replicates, at 12, 10 and 8 copies/μL.

The reproducibility of the HRM-RTqPCR assays for the detection of SARS-CoV-2 was also evaluated by the amplification (Ct values) of the synthetic sequences at 12, 10 and 8 copies/μL. Amplification was performed in 40 technical replicates for each template concentration, between two consecutive days, by the same operator. The box plot in Fig 2 shows the distribution of Ct values in the technical replicates and between the two days of analysis, for each target. For the N gene target, the median Ct values for days one and two, at 12 copies/μL, were respectively 29.57 and 29.89. At 10 copies/μL, the median Ct values were 30.46 and 30.34 and at 8 copies/μL, the median Ct values were 30.39 and 30.63, respectively. For the RdRp gene target, the median Ct values for days 1 and 2, at 12 copies/μL, were respectively 31.49 and 29.92. At 10 copies/μL, the median Ct values were 30.42 and 30.84 and, at 8 copies/μL, the median Ct values were 30.40 and 30.78, respectively. For the E target, the median Ct values for days one and two, at 12 copies/μL, were respectively 28.58 and 28.93. At 10 copies/μL, the median Ct values were 28.25 and 28.62 and, at 8 copies/μL, the median Ct values were 28.42 and 28.61, respectively. Some dispersion of Ct values in the replicates and a small number of outliers in all concentrations tested was observed. Additionally, no significant difference was observed in Ct values between day 1 and day 2 between all gene targets, reflecting good reproducibility of the HRM-qPCR assays.

### Clinical validation of HRM-RTqPCR assays

**Fig 3:**
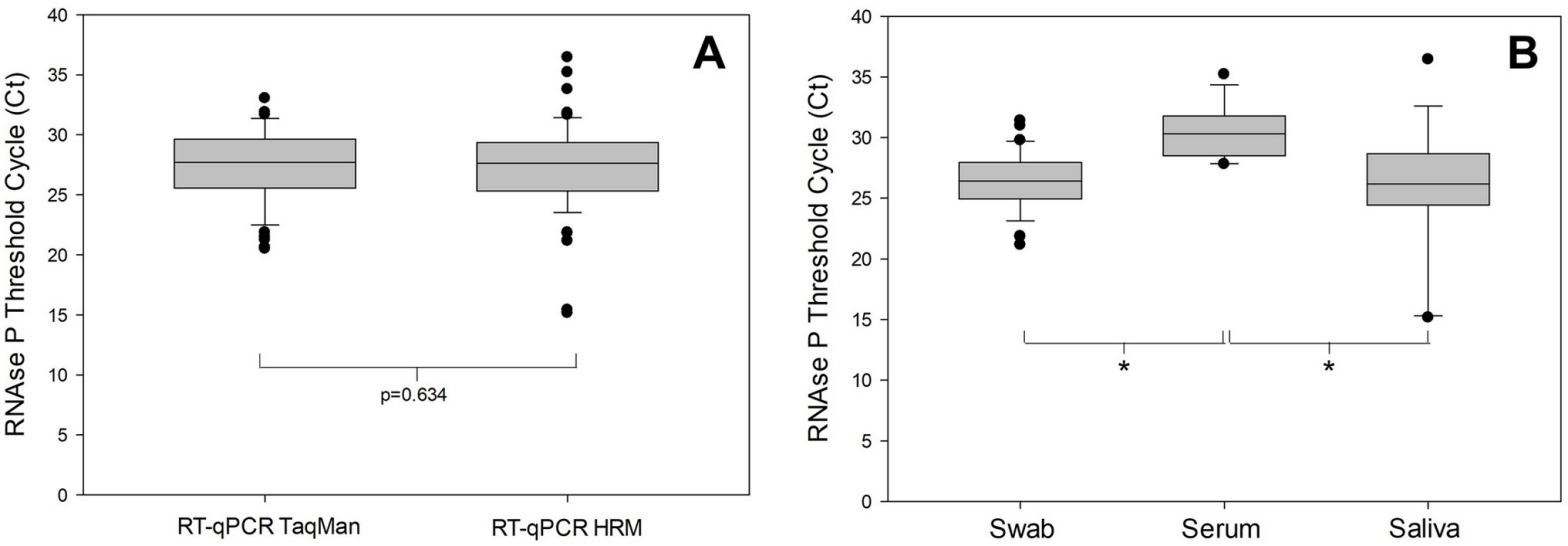
Boxplot of RNAse P Ct values between RT-qPCR TaqMan and RT-qPCR HRM assays and between swab, serum and saliva samples. The Ct values for the amplification of the internal control, human RNAse P, between TaqMan and HRM assays were compared between all the collected clinical specimens (A) or between swab, serum and saliva samples (B). The outliers are represented as the black circles. In A, p = 0.634 (Mann-Whitney Rank Sum Test). In B, *P<0.05 (One-way ANOVA) and p = 1.00 (Swab x Saliva—Mann-Whitney Rank Sum Test).

In order to assess the quality of the samples and RNA extraction, RNA concentration and purity was evaluated by the measurement of absorbance at 260 and 280 nm. The RNA concentration means (±Standard Deviation) were 40.55±36.50, 24.05±22.71 and 94.65±55.04 for swab, serum and saliva, respectively. All the RNA samples presented A260/280nm above 1.8. To differentiate between true- and false-negative results, the amplification of the internal control human RNAse P gene was monitored in all clinical specimens used in the TaqMan RT-qPCR and HRM-RTqPCR assays, in parallel to the detection of SARS-CoV-2 (Fig 3A). There was no significant difference in the observed Ct values of the TaqMan and HRM assays (p = 0.634), showing similar amplification of the RNAse P gene target in the HRM assay developed in this study. In addition, all the samples presented a Ct<35 for the RNAse P gene target, except for samples 54 (Ct 35.203 –serum) and 65 (Ct 36.440 –saliva) (S2 Table). It means an expected yield in RNA extraction and absence of PCR inhibition, except for samples 54 and 65. However, when the Ct values between the type of clinical samples (swab, serum or saliva–Fig 3B) were compared, significantly higher Ct values were observed in the serum samples compared to nasopharyngeal swab or saliva collected samples. No significant difference was observed between the nasopharyngeal swab and saliva samples (p = 1.000) (Fig 3B), indicating a similar RNA quantity and recovery between both type of samples.

After assessing the quality of the samples through the amplification of an internal control, human RNAse P, detection of SARS-CoV-2 was performed with samples collected from 65 patients, all residing in the Brazilian states of Rio de Janeiro and Ceará (42 swab, 12 serum and 11 saliva samples). The TaqMan assay which only targets the N gene of SARS-CoV-2 was considered the gold standard for SARS-CoV-2 detection. Of the 65 samples tested using the TaqMan assay, 51 were positive and 14 were negative; 97.6% of the swab, 16.7% of the serum, and 72.7% of the saliva samples were positive. The sensitivity of the HRM RTqPCR assays targeting the SARS-CoV-2 N, RdRp and E genes was respectively, 94.12, 98.04 and 92.16% (Table 3). Specificity to the 3 viral gene targets was 100% in the HRM-RTqPCR assay. The Positive Predictive Values was 100% to all targets, and the Negative Predictive Values were 82.35, 93.33 and 77.18% for the N, RdRp and E targets, respectively. In addition, the diagnostic accuracy was 95.38, 98.46 and 93.85%, respectively. To estimate the agreement between the HRM and TaqMan assays, the Cohen kappa coefficient was calculated. The coefficients of 0.87, 0.96 and 0.84 to the N, RdRp and E targets, were calculated respectively. The three HRM assays presented high clinical validation parameters, with an elevated diagnostic accuracy above 90%, in comparison to the TaqMan assay, including one positive amplification in serum samples (from 12) and 8 positive amplifications in saliva samples (from 11). The HRM assay targeting the RdRp gene showed the best clinical performance, with the highest sensitivity, specificity, PPV, NPV, accuracy, and Cohen’s kappa coefficient levels.

**Table 3:** Clinical validation of RT-qPCR assays targeting SARS-CoV-2 E, N and RdRp. The parameters of clinical validation were calculated considering RT-qPCR with TaqMan assays as gold standard, using the OpenEpi (v. 3) software.

Parameters	HRM RTqPCR assays		
	N target [CI 95%]	RdRp target [CI 95%]	E target [CI 95%]
**Samples number**	65	65	65
**Sensitivity (%)**	94.12 [84.08, 97.98]	98.04 [89.7–99.65]	92.16 [81.5–96.91]
**Specificity (%)**	100 [78.47, 100]	100 [78.47–100]	100 [78.47–100]
**PPV (%)**	100 [92.59, 100]	100 [92.86–100]	100 [92.44–100]
**NPV (%)**	82.35 [58.97, 93.81]	93.33 [70.18–98.81]	77.18 [54.78–91]
**Diagnostic accuracy (%)**	95.38 [87.29, 98.42]	98.46 [91.79–99.73]	93.85 [85.22–97.58]
**Cohen’s kappa coefficient**	0.87 [0.63–1.11]	0.96 [0.71–1.19]	0.84 [0.60–1.08]

PPV: Positive predictive value, NPV: Negative predictive value, CI95%: 95% Confidence Intervals.

**Fig 4:**
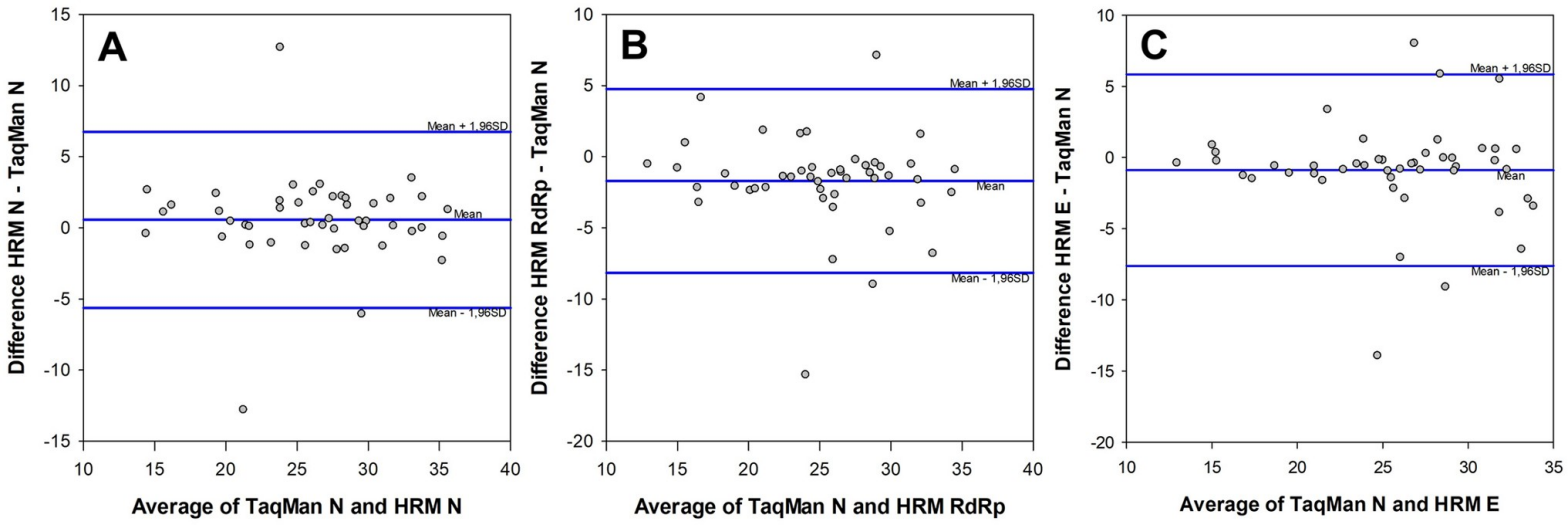
Bland-Altman plots resulting from the pairwise comparison of Ct values between RT-qPCR TaqMan and HRM-RTqPCR assays targeting SARS-CoV-2 N, RdRp and E. A. TaqMan N x HRM N targets. B. TaqMan N x HRM RdRp targets. C. TaqMan N x HRM E targets. Level of agreement between two assays, also known as observed mean difference (blue line at center), is presented as a function of the average Ct values of the two assays. Upper and lower limits of agreement (Upper and lower blue lines, respectively) defined by mean Ct difference ± 1.96 Standard Deviation (SD). The bias observed were 0.56, -1.71 and -0.89 to A, B and C, respectively.

The Bland-Altman Plot is a method to evaluate the agreement between two different assays, allowing the identification of any systematic difference between the measurements (i.e., fixed bias) or possible outliers. Thus, the pairwise comparison of Ct values between RT-qPCR TaqMan and HRM-RTqPCR assays to the N, RdRp and E targets in SARS-CoV-2 was performed using a Bland-Altman Plot (Fig 4). To the comparison between TaqMan and HRM to the N target for the 65 specimens analyzed, a bias of 0.56 and only three outliers (with Ct difference outside de mean±1.96 Standard Deviation) were observed (Fig 4A). When the comparison was performed between the TaqMan N and HRM RdRp assays, a bias of -1.71 with only three outliers was observed (Fig 4B). Lastly, when the comparison was between TaqMan N and HRM E assays, a bias of -0.89 with also three outliers were observed. In all comparisons, those parameters represent a high agreement between the assays, which resulted in a performance of the HRM-RTqPCR similar to the gold standard TaqMan RTqPCR assay.

## Discussion

With the emergence of the COVID-19 pandemic, the world faced an unprecedented need for RT-qPCR-based molecular diagnostic tests, reducing the availability of molecular diagnostic testing kits and related reagents, especially in developing countries. In March 2020, the WHO produced a technical manual recommending seven RT-qPCR assays developed and validated by globally recognized research centers to assist institutions, such as universities and small research groups, in providing assistance to the frontline of COVID-19 diagnostics testing [8]. However, with the increased demand for molecular diagnostic testing, the costs for commercial kits and reagents increased, contributing to the development of alternative methods for the detection of SARS-CoV-2 in clinical specimens. The main focus of this study was to develop a low-cost, non-commercial HRM-RTqPCR assay by using alternative methods and cost-effective reagents to aid in the diagnostic testing of COVID-19, especially in budget-restricted laboratories.

HRM analysis is a method developed in 2002 by the University of Utah (USA) and Idaho Technology (USA) for DNA sequence analysis [9]. Due to its ease in DNA genotyping, mutation screening, and sequence matching, the number of studies reporting its use continuously grows. The cost-advantage of HRM analysis results from the simplicity of its methodology: beyond the PCR reagents, it only requires a low-cost DNA saturating dye. Additionally, the typical time for a two-steps RT-qPCR assay with HRM is only 2.5 hours, an hour longer than a one-step RT-qPCR TaqMan assay. Although genotyping is the main application for an HRM analysis, the use of this methodology for the simultaneous molecular diagnostic and genotyping of pathogens, including the first Coronavirus, makes this a very promising technique [10, 13–15].

We first evaluated all the primers sequences *in silico*, selecting three primers pairs targeting the N, RdRp and E regions of SARS-CoV-2 genome. As the main objective of this study was to develop HRM-RTqPCR assays for the molecular detection of SARS-CoV-2, regardless of the accumulated mutations by the variants of concern, it was important to select the most stable genes for these diagnostic assays. By selecting the most stable genes to target for diagnostic assays, we avoid the presence of insertions, deletions or SNPs that can lead to primer mismatches or differences in the shape of the HRM curves, which would be used to certify the specificity of the RT-qPCR amplifications.

In this study, the analytical validation of HRM-qPCR assays was performed using international guidelines [16] and synthetic sequence templates of the respective SARS-CoV-2 genes. Our first results showed a sharp peak for each of the SARS-CoV-2 gene targets and the human RNAse P gene, at the derivative melting curves, with Tms differing at least 2.5°C between the targets. Although it was not explored in this study, the Tm differences open a possibility for multiplexing these assays, providing an opportunity in lowering molecular diagnostic testing costs. However, when developing a multiplexing assay, it would be necessary to standardize the assays by modifying the concentrations of primers and master mix reagents, as MgCl_2_ and dNTPs, which is limited in the available commercial HRM master mixes.

The observed dynamic range of the HRM-RTqPCR assays to the 3 SARS-CoV-2 gene targets presented 5 logs and approximated LOD of 10 copies/μL, with PCR efficiencies between 88.3% and 109.7%. The shape of the normalized HRM curves was very similar between all positive patient samples and the synthetic control templates, used as reference. In fact, the clinical specificity observed within those three HRM-RTqPCR assays was 100%, which corresponds to the similarity in shape and peak of each HRM curve. If a mutation occurred in the N, RdRp and E regions amplified by the primers used, it would be possible to observe a difference in the normalized HRM curves, which would be detected by the HRM software and reported as a variation. In this study, the 145 positive HRM-RTqPCR results, with similar shapes in the normalized HRM curves, suggest the stability of the N, RdRp and E regions in the SARS-CoV-2 genome within these samples.

One of the main concerns when repeating HRM-based assays has been their repeatability and reproducibility. In accordance with the Clinical and Laboratory Standards Institute (CLSI) document EP12-A2 for qualitative tests [17], we performed a replication experiment to evaluate precision of the three HRM-RTqPCR assays to SARS-CoV-2 using three low concentrations of the synthetic control templates, around the estimated LOD. The parameters of precision were evaluated using the detected Ct values from each assay. The three assays presented a high precision, estimated by the observed small coefficient of variation which was below 2%, except for the E gene target assay at 8 copies/μL (3.65%). The CV, however, was below the recommended 15% value in this previously mentioned gene target [18]. To evaluate reproducibility, three assays were performed in twenty technical replicates for each concentration around the LOD, by the same operator in two consecutive days, for a total of forty replicates. The Ct values were compared between day one and day two for each target, but no statistical difference was observed (Fig 2), validating the reproducibility of this method.

The clinical validation for the HRM-RTqPCR assays was performed with 65 samples from patients of the Rio de Janeiro and Ceará States in Brazil, mostly nasopharyngeal swabs, but also saliva and serum. The differences in clinical specimens and origin of the patients were relevant to enrich the methodology validation for different scenarios, including the possibility of different SARS-CoV-2 variants, their detection, and the difference in sensitivity of samples with differing viral loads. The human RNAse P gene was used as internal control, for both TaqMan and HRM assays. No statistical difference was observed in the RNAse P Ct values between TaqMan and HRM assays, suggesting that the sensitivity for this target was similar between the two methods. Nevertheless, when the Ct values were compared between the different type of samples in the HRM-RTqPCR assays, serum samples presented higher RNAse P Ct values, which was significantly different from that observed in nasopharyngeal swabs and saliva samples. As expected, during serum preparation, cells containing mRNA, including the RNAse P transcript, could be trapped within the clot. This could result in a decreased detection of this human gene target in serum samples. Regardless, previous studies already described detection of SARS-CoV-2 in serum [19], which could be associated with increased mortality risk in hospitalized COVID-19 patients [20].

As reported, the three HRM assays validated herein presented a high sensitivity (from 92.16% to 98,04%) and specificity (all 100%). Regardless of the sample type (serum, saliva or nasopharyngeal swab), a very good concordance could be observed with the TaqMan assay, except for three nasopharyngeal swab and one serum samples (S2 Table). From those, only one sample was positive using TaqMan assay but negative in all three HRM assays. This sample presented Ct = 37.02±0.07, the highest Ct observed to the TaqMan assays in this study. In addition, the other three samples also presented high Ct values to the TaqMan assay, higher than 34. It is important to mention that most patients suspected of having COVID-19 seek medical helping during the acute phase of the disease, in which a high viral load is observed, and molecular diagnostic testing is recommended [21]. In addition, the observed Ct value variability among the positive samples can be attributed to collection of the samples at unknown time points between viral infection and symptom onset. However, other variables such as patient age and the clade of the virus could contribute to the observed differences. The HRM-RTqPCR assays validated in this study presented an increased sensitivity for the detection of the SARS-CoV-2 targeted genes considering the samples contained lower viral loads; the observation of lowered viral loads was made from the high Ct values observed in the gold standard assay.

Finally, the agreement between TaqMan and HRM assays was evaluated by the Bland-Altman plot analysis with the pairwise comparison of Ct values. The HRM assays to three targets in the virus were compared to the TaqMan assay targeting the N region. The three HRM assays present a high agreement with the TaqMan assay, with biases very close to zero and only three outliers (out of ±1.96 Standard Deviation) in each comparison. It means that the Ct values are very similar performing the TaqMan or HRM assays, even for different targets in the SARS-CoV-2 genome. Considering that each region presents only one copy at the virus genome, and the PCR efficiencies were very similar among the targets and close to 100%, it is plausible to expect similar Ct values to all the targets and the TaqMan assay. Taken together, our results open a new venue of low-cost alternatives for the molecular diagnostic of COVID-19 in differently acquired samples, with the promising application of SARS-CoV-2 genotyping and identification of variants of concern, especially for the detection of mutations on the SARS-CoV-2 S gene as our developed approach could be adapted to the S region of the viral genome. Of note, the estimated cost for the HRM-RTqPCR assay validated herein was US$ 10 per sample, a cost considerably lower than any commercial kit available for the molecular diagnostic testing of COVID-19 including TaqMan-based systems and conventional DNA sequencing used to identify SARS-CoV-2 variants. Altogether, SYBR-Green and HRM-based methodologies are emerging in the literature as promising alternatives to the molecular diagnostics of COVID-19 and identification of SARS-CoV-2 variants of concern [22–25].

Overall, the main strength of this study was the development and validation of an alternative methodology for the molecular diagnosis of COVID-19, with high sensitivity, specificity, reproducibility, and economic advantages in comparison to the gold standard assay. Regarding the study limitations, the three molecular targets used did not allow the identification of the SARS-CoV-2 variants of concern, which would be an important use for a RT-qPCR with high resolution melting methodology. However, based on the assays developed herein, we are now working on the validation of HRM assays targeting different regions of the SARS-CoV-2 S gene, to simultaneously detect and discriminate the variants of concern, which will be an important advance to the molecular diagnosis of COVID-19.

## Conclusion

The HRM-RTqPCR assays targeting the N, RdRp and E regions of the SARS-CoV-2 presented high sensitivity and specificity to the molecular diagnostic of COVID-19 from nasopharyngeal swabs, serum or saliva samples, using a low-cost alternative to the TaqMan assays currently available and offering a promising approach for the discrimination of the SARS-CoV-2 variants of concern.

## Supporting information

S1 Table Synthetic single-strand DNA templates from SARS-CoV-2 and human genome used in this study. (DOCX)

S2 Table Validation of the HRM-RTqPCR assays for SARS-CoV-2 E, N and RdRp targets. Sixty-five RNA samples were extracted from nasopharyngeal swab, serum or saliva of patients suspicious for COVID-19. All samples were analyzed for RT-qPCR TaqMan assays targeting SARS-CoV-2 N2 and Human RNAse P targets, in parallel to the HRM-RTqPCR assays.
(DOCX)
