# Supplementary material for: Validation of a novel molecular assay to the diagnostic of COVID-19 based on real time PCR with high resolution melting
Source: PLoS One. 2021 Nov 22;16(11):e0260087. doi: 10.1371/journal.pone.0260087 (PMC8608302; doi:10.1371/journal.pone.0260087)
Supplement: S1 Table — (DOCX) [file pone.0260087.s001.docx]

**Table S1. Synthetic single-strand DNA templates from SARS-CoV-2 and human genome used in this study.**

| **Target** | **Sequences**  **(5’ – 3’)** | **Description** | **Amplicon size** | **Access number**  **(GenBank)** |
| --- | --- | --- | --- | --- |
| **N** | GGGACCAGGAACTAATCAGACAAGGAACTGATTACAAACATTGGCCGCAAATTGCACAATTTGCCCCCAGCGCTTCAGCGTTCTTCGGAATGTCGCGCATTGGCATGGAAGTCACACCTTCGGGA | Severe acute respiratory syndrome coronavirus 2 isolate Wuhan-Hu-1, complete genome | 125 bp | NC_045512.2 |
| **RdRp** | ATCTCAATGGTAACTGGTATGATTTCGGTGATTTCATACAAACCACGCCAGGTAGTGGAGTTCCTGTTGTAGATTCTTATTATTCATTGTTAATGCCTATATTAACCTTGACCAGGGCTTTAACT | Severe acute respiratory syndrome coronavirus 2 isolate Wuhan-Hu-1, complete genome | 125 bp | NC_045512.2 |
| **E** | GAAGAGACAGGTACGTTAATAGTTAATAGCGTACTTCTTTTTCTTGCTTTCGTGGTATTCTTGCTAGTTACACTAGCCATCCTTACTGCGCTTCGATTGTGTGCGTACTGCTGCAATATTGTTAA | Severe acute respiratory syndrome coronavirus 2 isolate Wuhan-Hu-1, complete genome | 125 bp | NC_045512.2 |
| **RNAse P** | CAGCATGGCGGTGTTTGCAGATTTGGACCTGCGAGCGGGTTCTGACCTGAAGGCTCTGCGCGGACTTGTGGAGACAGCCGCTCACCTTGGCTATTCAGTT | Homo sapiens ribonuclease P/MRP subunit p30 (RPP30), transcript variant 2, mRNA | 100 bp | NM_006413.5 |
