# Supplementary material for: Validation of a novel molecular assay to the diagnostic of COVID-19 based on real time PCR with high resolution melting
Source: PLoS One. 2021 Nov 22;16(11):e0260087. doi: 10.1371/journal.pone.0260087 (PMC8608302; doi:10.1371/journal.pone.0260087)
Supplement: S2 Table — Sixty-five RNA samples were extracted from nasopharyngeal swab, serum or saliva of patients suspicious for COVID-19. All samples were analyzed for RT-qPCR TaqMan assays targeting SARS-CoV-2 N2 and Human RNAse P targets, in parallel to the HRM-RTqPCR assays. (DOCX) [file pone.0260087.s002.docx]

**Table S2. Validation of the HRM-RTqPCR assays for SARS-CoV-2 E, N and RdRp targets.** Sixty-five RNA samples were extracted from nasopharyngeal swab, serum or saliva of patients suspicious for Covid-19. All samples were analyzed for RT-qPCR TaqMan assays targeting SARS-CoV-2 N2 and Human RNAseP targets, in parallel to the HRM-RTqPCR assays.

| # | **Sample** | **TaqMan RNAse P target** | | | **TaqMan N target** | | | **HRM N target** | | | **HRM RdRp target** | | | **HRM E target** | | | **HRM Rnase P target** | | |
| --- | --- | --- | --- | --- | --- | --- | --- | --- | --- | --- | --- | --- | --- | --- | --- | --- | --- | --- | --- |
|  |  | **Result** | **CT Mean** | **Ct SD** | **Result** | **CT Mean** | **Ct SD** | **Result** | **CT Mean** | **Ct SD** | **Result** | **CT Mean** | **Ct SD** | **Result** | **CT Mean** | **Ct SD** | **Result** | **CT Mean** | **Ct SD** |
| **1** | Swab | Positive | 22.532 | 0.001 | Positive | 36.360 | 0.640 | Positive | 34.046 | 0.295 | Positive | 29.556 | 0.133 | Positive | 29.907 | 1.809 | ND | - | - |
| **2** | Swab | Positive | 27.511 | 0.087 | Positive | 34.974 | 0.442 | Positive | 36.243 | 2.722 | Positive | 34.065 | 1.978 | Positive | 32.057 | 1.953 | ND | - | - |
| **3** | Swab | Positive | 26.887 | 0.189 | Positive | 25.448 | 0.128 | Positive | 25.714 | 0.445 | Positive | 32.564 | 0.610 | Positive | 31.302 | 1.516 | ND | - | - |
| **4** | Swab | Positive | 26.403 | 0.107 | Positive | 29.113 | 0.016 | Positive | 27.656 | 0.135 | Positive | 27.978 | 0.568 | Positive | 34.611 | 5.015 | ND | - | - |
| **5** | Swab | Positive | 25.485 | 0.004 | Positive | 33.784 | 1.062 | Positive | 33.780 | 1.193 | Positive | 30.510 | 0.362 | Positive | 29.909 | 1.855 | ND | - | - |
| **6** | Swab | Positive | 23.845 | 0.000 | Positive | 32.576 | 0.385 | Positive | 26.523 | 0.172 | Positive | 27.311 | 0.518 | Positive | 33.135 | 1.803 | ND | - | - |
| **7** | Swab | Positive | 25.603 | 0.020 | Positive | 31.675 | 6.020 | Positive | 30.389 | 0.049 | Positive | 16.340 | 0.417 | Positive | 17.745 | 0.409 | ND | - | - |
| **8** | Swab | Positive | 24.271 | 0.072 | Positive | 29.563 | 0.213 | Positive | 31.246 | 0.227 | Positive | 22.319 | 0.048 | Positive | 22.535 | 0.071 | ND | - | - |
| **9** | Swab | Positive | 24.857 | 0.008 | Positive | 22.852 | 0.033 | Positive | 24.752 | 0.004 | Positive | 24.463 | 0.101 | Positive | 30.872 | 0.225 | ND | - | - |
| **10** | Swab | Positive | 23.919 | 0.120 | Positive | 33.239 | 0.510 | Positive | 32.974 | 1.156 | Positive | 24.262 | 0.561 | Positive | 24.137 | 0.454 | ND | - | - |
| **11** | Swab | Positive | 24.894 | 0.052 | Positive | 26.734 | 0.232 | Positive | 26.911 | 0.304 | Positive | 23.785 | 0.014 | Positive | 24.566 | 0.217 | Positive | 23.701 | 0.166 |
| **12** | Swab | Positive | 27.726 | 0.105 | Positive | 27.744 | 0.215 | Positive | 29.333 | 0.092 | Positive | 24.172 | 0.343 | Positive | 24.863 | 0.059 | Positive | 27.677 | 0.143 |
| **13** | Swab | Positive | 28.674 | 0.290 | Positive | 25.786 | 0.227 | Positive | 26.149 | 0.259 | Positive | 24.022 | 0.203 | Positive | 24.840 | 0.179 | Positive | 27.926 | 0.038 |
| **14** | Swab | Positive | 25.630 | 0.080 | Positive | 26.227 | 0.595 | Positive | 24.959 | 0.266 | Positive | 23.912 | 0.041 | Positive | 24.800 | 0.080 | Positive | 24.612 | 0.149 |
| **15** | Swab | Positive | 25.538 | 0.085 | Positive | 28.586 | - | Positive | 27.051 | 0.352 | Positive | 27.943 | 0.729 | Positive | 28.555 | 0.633 | Positive | 24.404 | 0.024 |
| **16** | Swab | Positive | 30.986 | - | Positive | 24.255 | 0.009 | Positive | 26.002 | 0.813 | Positive | 23.235 | 0.077 | Positive | 23.665 | 0.097 | Positive | 30.972 | 0.209 |
| **17** | Swab | Positive | 30.358 | 0.972 | Positive | 25.109 | 0.061 | Positive | 28.161 | 0.415 | Positive | 23.650 | 0.183 | Positive | 24.917 | 0.347 | Positive | 29.348 | 0.493 |
| **18** | Swab | Positive | 29.502 | - | Positive | 20.101 | 0.081 | Positive | 20.561 | 0.202 | Positive | 21.956 | 0.497 | Positive | 23.463 | 1.987 | Positive | 29.687 | 0.073 |
| **19** | Swab | Positive | 30.780 | 0.188 | Positive | 27.628 | - | Positive | 14.835 | 0.701 | Positive | 27.417 | 0.313 | Positive | 28.853 | 0.748 | Positive | 31.393 | - |
| **20** | Swab | Positive | 25.132 | 0.034 | Positive | 17.483 | 0.703 | Positive | 30.163 | 0.797 | Positive | 15.310 | 0.090 | Positive | 16.209 | 0.092 | Positive | 24.254 | 0.028 |
| **21** | Swab | Positive | 27.585 | 0.075 | Positive | 26.454 | 0.087 | Positive | 28.627 | 0.133 | Positive | 25.269 | 0.032 | Positive | 25.629 | 0.177 | Positive | 27.758 | 0.304 |
| **22** | Swab | Positive | 27.700 | 0.012 | Positive | 27.046 | 0.195 | Positive | 29.271 | 0.185 | Positive | 25.959 | 0.003 | Positive | 26.646 | 0.430 | Positive | 27.965 | 0.431 |
| **23** | Swab | Positive | 26.404 | 0.095 | Positive | 23.727 | 0.243 | Positive | 22.661 | 0.217 | Positive | 22.281 | 0.055 | Positive | 23.268 | 0.145 | Positive | 25.554 | 0.138 |
| **24** | Swab | Positive | 29.623 | 0.050 | Positive | 29.124 | 0.266 | Positive | 29.591 | 0.009 | Positive | 28.683 | 0.113 | Positive | 29.067 | 0.337 | Positive | 29.769 | 0.137 |
| **25** | Swab | Positive | 28.550 | 0.003 | Positive | 29.653 | 0.336 | Positive | 30.110 | 0.603 | Positive | 28.105 | 0.406 | Positive | 28.984 | 0.372 | Positive | 28.406 | 0.074 |
| **26** | Swab | Positive | 27.749 | 0.042 | Positive | 21.306 | 0.065 | Positive | 21.494 | 0.160 | Positive | 18.930 | 0.120 | Positive | 20.687 | 0.122 | Positive | 27.582 | 0.016 |
| **27** | Swab | Positive | 26.435 | 0.021 | Positive | 22.300 | 0.014 | Positive | 21.089 | 0.274 | Positive | 20.125 | 0.349 | Positive | 20.672 | 0.083 | Positive | 26.233 | 0.773 |
| **28** | Swab | Positive | 27.285 | 0.068 | Positive | 27.674 | 0.317 | Positive | 27.588 | 0.223 | Positive | 26.133 | 0.513 | Positive | 26.781 | 0.640 | Positive | 27.119 | 0.346 |
| **29** | Swab | Positive | 28.108 | 0.602 | Positive | 20.086 | 0.126 | Positive | 19.432 | 0.275 | Positive | 18.003 | 0.323 | Positive | 18.986 | 0.302 | Positive | 27.004 | 0.321 |
| **30** | Swab | Positive | 27.649 | 0.099 | Positive | 21.600 | 0.049 | Positive | 21.699 | 0.041 | Positive | 19.336 | 0.004 | Positive | 20.432 | 0.108 | Positive | 28.094 | 0.801 |
| **31** | Swab | Positive | 25.415 | 0.041 | Positive | 32.719 | 0.503 | Positive | 34.895 | 0.992 | Positive | 31.092 | 0.414 | Positive | 31.852 | 1.908 | Positive | 25.381 | 0.335 |
| **32** | Swab | Positive | 20.494 | 0.039 | Positive | 14.594 | 0.013 | Positive | 14.185 | 4.881 | Positive | 18.742 | 1.879 | Positive | 15.471 | 0.381 | Positive | 21.810 | 0.458 |
| **33** | Swab | Positive | 26.169 | 0.004 | Positive | 36.124 | 0.016 | Negative | - | - | Positive | 33.856 | 1.787 | Negative | - | - | Positive | 26.438 | 0.626 |
| **34** | Swab | Positive | 25.722 | 0.007 | Positive | 34.495 | 1.353 | Negative | - | - | Positive | 24.669 | 2.558 | Negative | - | - | Positive | 25.659 | 0.260 |
| **35** | Swab | Positive | 26.371 | 0.058 | Positive | 34.311 | 1.256 | Positive | 34.629 | 0.951 | Positive | 35.112 | 1.989 | Negative | - | - | Positive | 26.192 | 0.029 |
| **36** | Swab | Positive | 21.868 | 0.253 | Positive | 13.158 | 0.025 | Positive | 15.822 | 0.045 | Positive | 12.639 | 0.927 | Positive | 12.762 | 1.618 | Positive | 21.855 | 0.481 |
| **37** | Swab | Positive | 22.558 | 0.005 | Positive | 18.980 | 0.021 | Positive | 20.133 | 0.170 | Positive | 17.761 | 0.414 | Positive | 18.386 | 0.276 | Positive | 26.773 | 5.064 |
| **38** | Swab | Positive | 25.281 | 0.114 | Positive | 35.553 | 2.265 | Positive | 34.949 | 0.252 | Positive | 33.027 | 0.346 | Positive | 32.124 | - | Positive | 26.263 | 1.759 |
| **39** | Swab | Positive | 25.983 | 0.077 | Positive | 30.551 | 0.006 | Positive | 32.607 | 0.880 | Positive | 29.192 | 0.235 | Positive | 31.159 | 0.821 | Positive | 26.400 | 0.179 |
| **40** | Swab | Positive | 25.214 | 0.025 | Positive | 31.323 | 0.044 | Positive | 34.824 | 0.480 | Positive | 32.893 | 0.745 | Positive | 31.909 | 0.442 | Positive | 25.256 | 0.045 |
| **41** | Swab | Positive | 21.479 | 0.085 | Positive | 18.116 | 0.047 | Positive | 20.528 | 1.098 | Positive | 14.890 | 0.166 | Positive | 16.619 | 1.909 | Positive | 21.168 | 0.058 |
| **42** | Swab | Positive | 24.264 | 0.040 | Negative | - | - | Negative | - | - | Negative | - | - | Negative | - | - | Positive | 24.383 | 0.199 |
| **43** | Serum | Positive | 28.144 | 0.085 | Positive | 31.705 | 0.243 | Positive | 31.849 | 0.202 | Positive | 31.178 | 0.036 | Positive | 31.478 | 0.959 | Positive | 28.369 | 0.874 |
| **44** | Serum | Positive | 34.892 | 0.356 | Negative | - | - | Negative | - | - | Negative | - | - | Negative | - | - | ND | - | - |
| **45** | Serum | Positive | 31.867 | 0.490 | Negative | - | - | Negative | - | - | Negative | - | - | Negative | - | - | Positive | 30.630 | 1.562 |
| **46** | Serum | Positive | 28.840 | 0.004 | Negative | - | - | Negative | - | - | Negative | - | - | Negative | - | - | Positive | 28.957 | 0.031 |
| **47** | Serum | Positive | 29.816 | 0.339 | Negative | - | - | Negative | - | - | Negative | - | - | Negative | - | - | Positive | 30.312 | 0.009 |
| **48** | Serum | Positive | 31.689 | 0.012 | Negative | - | - | Negative | - | - | Negative | - | - | Negative | - | - | Positive | 31.647 | 0.015 |
| **49** | Serum | Positive | 29.690 | 0.467 | Negative | - | - | Negative | - | - | Negative | - | - | Negative | - | - | Positive | 29.496 | 0.269 |
| **50** | Serum | Positive | 33.030 | 0.005 | Negative | - | - | Negative | - | - | Negative | - | - | Negative | - | - | Positive | 31.822 |  |
| **51** | Serum | Positive | 28.146 | 0.028 | Negative | - | - | Negative | - | - | Negative | - | - | Negative | - | - | Positive | 27.801 | 0.499 |
| **52** | Serum | Positive | 27.841 | 0.179 | Negative | - | - | Negative | - | - | Negative | - | - | Negative | - | - | Positive | 27.914 | 0.320 |
| **53** | Serum | Positive | 25.791 | 0.186 | Positive | 37.021 | 0.067 | Negative | - | - | Negative | - | - | Negative | - | - | Positive | 33.781 |  |
| **54** | Serum | Positive | 31.327 | 0.441 | Negative | - | - | Negative | - | - | Negative | - | - | Negative | - | - | Positive | 35.203 | 5.319 |
| **55** | Saliva | Positive | 40.481 |  | Negative | **-** | - | Negative | - | - | Negative | - | - | Negative | - | - | Positive | 30.043 | 2.957 |
| **56** | Saliva | Positive | 29.585 | 0.086 | Positive | 24.878 | 0.063 | Positive | 27.402 | 1.252 | Positive | 24.103 | 0.273 | Positive | 24.715 | 0.328 | Positive | 25.307 | 0.063 |
| **57** | Saliva | Positive | 27.635 | 0.332 | Positive | 15.403 | 0.138 | Positive | 16.991 | 0.688 | Positive | 14.607 | 0.933 | Positive | 15.157 | 0.230 | Positive | 15.157 | 0.230 |
| **58** | Saliva | Positive | 31.120 | 0.309 | Positive | 29.670 | 0.308 | Positive | 29.752 | 0.053 | Positive | 28.951 | 0.463 | Positive | 28.722 | 1.346 | Positive | 28.722 | 1.346 |
| **59** | Saliva | Positive | 31.874 | 0.290 | Positive | 23.130 | 0.244 | Positive | 24.508 | 0.161 | Positive | 21.731 | 0.018 | Positive | 22.276 | 0.745 | Positive | 24.400 | 1.991 |
| **60** | Saliva | Positive | 29.052 | 0.118 | Positive | 26.943 | 0.009 | Positive | 27.583 | 0.175 | Positive | 25.997 | 0.576 | Positive | 26.484 | 0.523 | Positive | 26.192 | 1.580 |
| **61** | Saliva | Positive | 29.442 | 0.184 | Negative | - | - | Negative | - | - | Negative | - | - | Negative | - | - | Positive | 28.529 | 4.730 |
| **62** | Saliva | Positive | 20.619 | 0.090 | Positive | 15.065 | 0.477 | Positive | 16.165 | 0.596 | Positive | 16.037 | 0.328 | Positive | 15.409 | 0.231 | Positive | 15.409 | 0.231 |
| **63** | Saliva | Positive | 22.808 | 0.167 | Positive | 27.407 | 0.078 | Positive | 29.484 | 0.279 | Positive | 24.730 | 2.410 | Positive | 27.680 | 1.011 | Positive | 28.005 | 0.224 |
| **64** | Saliva | Positive | 21.269 | 0.037 | Positive | 23.247 | 0.176 | Positive | 26.250 | 0.109 | Positive | 24.978 | 0.879 | Positive | 24.526 | 0.131 | Positive | 24.526 | 0.131 |
| **65** | Saliva | Positive | 30.843 | 0.391 | Negative | - | - | Negative | - | - | Negative | - | - | Negative | - | - | Positive | 36.440 | 4.027 |

ND: Not done. Ct: Threshold cycle. SD: Standard Deviation.
